# Multi-parameter enhanced optical encryption with biphasic chiral photonic crystals

**DOI:** 10.1038/s41377-026-02360-z

**Published:** 2026-06-04

**Authors:** Cheng Ouyang, Quanming Chen, Dewei Zhang, Zhiyao Xie, Dan Luo, Yan-qing Lu, Wei Hu

**Affiliations:** 1 National Laboratory of Solid State Microstructures, Jiangsu Physical Science Research Center, College of Engineering and Applied Sciences, Nanjing University, Nanjing, 210023 China; 2 Department of Electrical & Electronic Engineering, Southern University of Science and Technology, Shenzhen, 518055 China

**Keywords:** Liquid crystals, Optoelectronic devices and components

## Abstract

Encoding information across multiple degrees of light, including spin, wavelength, amplitude, and phase into the multi-level structures of a stimuli-responsive material, presents a highly promising strategy for optical encryption. Here, we present a biphasic chiral photonic crystal platform that addresses the intrinsic coupling among photonic spin, wavelength, and functions, thus providing a multi-parameter security framework that substantially enhances encryption complexity. By integrating two separately photopatternable chiral photonic crystals with opposite handedness into a single cell, independent geometric phase modulation for orthogonal spins and discrete wavelengths is fully released. The near-field polarization interference imaging and far-field spin-multiplexed holography with partly temperature-robust and partly thermally responsive information are demonstrated. Furthermore, we concealed the latitude and longitude coordinates of a destination across two separate far-field images, which are only revealed at the correct combination of temperature, optical spin, and wavelength. This biphasic system fully harnesses light’s potential for advanced encryption, which will drastically enhance the security of secure logistics, anti-counterfeiting, and hardware authentication.

## Abstract (web summary)

*Multi-parameter optical encryption* is demonstrated by spin- and wavelength-selective manipulation of both near-field imaging and holographic decoding in oppositely handed chiral photonic crystals.

## Graphical abstract

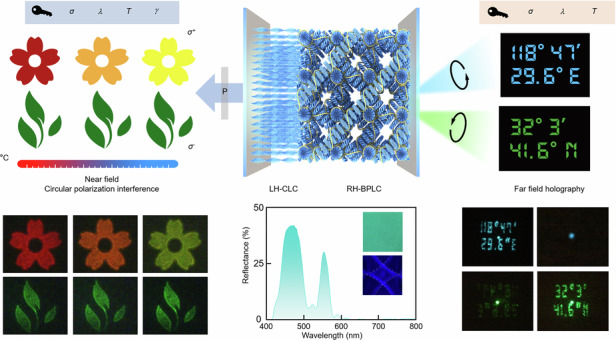

## Introduction

In the current digital era, driven by cloud storage, the Internet of Things (IoT), and artificial intelligence, information security remains a paramount global concern^1^. Conventional electronic encryption, which relies on computational complexity, faces a fundamental threat from rapidly growing computing power, particularly the advent of quantum computing^2,3^. In contrast, physical-layer security strategies like optical encryption offer inherent resistance to algorithmic attacks by leveraging light’s capacity for high-dimensional parallel processing^4^. This has spurred urgent demand for compact, robust, and low-cost optical solutions to serve emerging markets in secure logistics, high-value anti-counterfeiting^5^, privacy-preserving displays for sensitive sectors^6^, encrypted AR/VR systems^7^, and hardware-level authentication for the IoT^8^. To meet the demands of these diverse applications, it is especially significant to explore new photonic media capable of simultaneously and dynamically manipulating multiple light parameters, thereby fully harnessing the high-dimensional potential of light for advanced encryption.

Photonic crystals (PCs), characterized by their spatially periodic nanostructures, are pivotal in optical encryption due to their angle-dependent, narrow-band Bragg reflection^9^. Their utility is further enhanced by a responsiveness to external stimuli, which allows for dynamic tuning of their photonic bandgap and reflectance^10,11^. However, most previous works leveraging such structures only rely on the modulation of a single degree of freedom of light, including amplitude and wavelength^12,13^. While introducing more degrees is expected to significantly enhance the unclonability of information entropy. Therefore, it is very significant to bring circular polarization independent or directionally separated functions into optical encryptions and anti-counterfeiting techniques. Cholesteric liquid crystals (CLCs) offer a compelling solution, as they spontaneously form a one-dimensional (1D) chiral PC^14^. Their structure provides intrinsic circular polarization selectivity within the photonic bandgap^15^. By pre-setting the CLC’s helical alignment through photoalignment, a Bragg-Berry phase can be encoded into the reflected light^16^. Furthermore, the working band of CLCs can be customized by thermally^17^, optically^18^, or electrically^19^ tuning their helical pitch. In contrast, blue-phase liquid crystals (BPLCs), composed of cubic lattices of double-twisted cylinders, constitute a three-dimensional (3D) PC^20,21^. Beyond an angle-dependent bandgap, they can omnidirectionally encode a Bragg-Berry phase into reflected light, facilitating wide-angle polarization gratings, vector beam generators, and reflective holograms^22–24^. These unique properties make both CLCs and BPLCs exceptional media for multi-dimensional light manipulation. Strategies such as electrically driven gradient-pitch tuning^25^ and photo-stimulated chiral inversion^26^ now allow for precise tailoring of the chroma and spin of selectively reflected light. Meanwhile, cascaded layers^27^ and biphasic composites^28–30^ are being developed to decouple phase modulation for different wavelengths and spins. The integration of 1D CLC and 3D BPLC PCs is poised to drastically enhance the multichannel capability of optical encryption systems, making research in this area highly significant.

We present a biphasic chiral PCs platform that enables high-capacity, multichannel optical encryption. This system simultaneously generates distinct near-field and far-field optical information. In the near field, two separate images are generated via reflective circular-polarization interference. In the far field, independent holograms from each phase corresponding to specific circular polarization and wavelength are formed. The platform is realized by washing out the unreacted monomers and then refilling an oppositely-handed CLC into a polymer-stabilized BPLC (PS-BPLC). This process yields two stable, co-existing PCs: the original BPLC and a new CLC layer filled in the atmospheric gap created by network shrinkage. Crucially, the BPLC layer is thermally robust, while the CLC layer’s optical properties are temperature-sensitive. This multi-stimuli-responsiveness, combined with the orthogonal optical responses of the two phases, means hidden information can only be retrieved at the correct temperature, polarization, and wavelength. Our design not only drastically improves encryption security but also offers a versatile platform for advanced planar optics.

## Results

### Multichannel optical encryption via photopatterned biphasic chiral photonic crystals

**Fig. 1:**
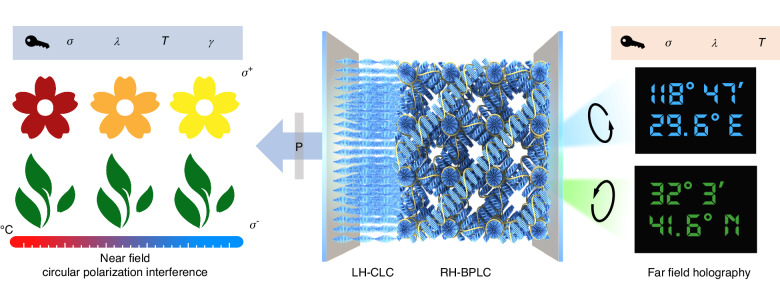
Schematic of multichannel optical encryption using photopatterned biphasic chiral photonic crystals. *The center schematic* illustrates the biphasic structure, comprising a cascaded LH-CLC and a PS-RH-BPLC, where yellow filaments and blue rods denote the polymer network and LC director, respectively. *On the left*, near-field images from circular-polarization interference show a flower and leaves encoded in the respective LH-CLC and RH-BPLC channels. The information is decoded across multiple physical parameters: *σ* (circular polarization), *λ* (wavelength), *T* (temperature), and *γ* (orientation of the analyzer). *On the right*, far-field spin-decoupled geometric-phase holography projects independent coordinate patterns (e.g., longitude and latitude) under illumination with orthogonal circular polarizations at their respective photo, utilizing *σ*, *λ*, and *T* as decryption keys

Figure 1 schematically illustrates the biphasic chiral PCs, a stack of a left-handed CLC (LH-CLC) and a polymer-stabilized right-handed BPLC (PS-RH-BPLC). Their opposite handedness grants orthogonal spin (*σ*) selectivity, while their distinct band gaps and separately photopatterned alignments (*θ*) permit wavelength (*λ*) as well as shape control over near-field reflection and far-field diffraction. In the near-field channel (left panel of Fig. 1), the circular polarization component matching the handedness and bandgap of each layer is reflected, acquiring a geometric phase twice the value of the local alignment direction. This phase-modulated reflection interferes with the reflected opposite circular polarization, and the recombined linear polarization forms the objective “flower” and “leaves” patterns when viewed through an analyzer. Notably, rotating the analyzer (*γ*) by 90° switches the images to their complementary versions. Simultaneously, the far-field channel (right panel of Fig. 1) projects distinct holographic images (e.g., longitude/latitude grids) under specific *σ* and *λ*. The optical information recorded in the PS-RH-BPLC layer can only be decoded by the correct *σ* and *λ*, and exhibits high thermal stability owing to the polymer stabilization. In contrast, the helical pitch of the CLC is temperature (*T*)-dependent, enabling thermal tuning of its operational wavelength. By leveraging these multiple physical channels (*σ*, *λ*, *T*, *γ*, *θ*) for information encoding, this design establishes a foundation for high-capacity optical encryption based on biphasic chiral PCs.

### Fabrication of biphasic chiral photonic crystals

**Fig. 2:**
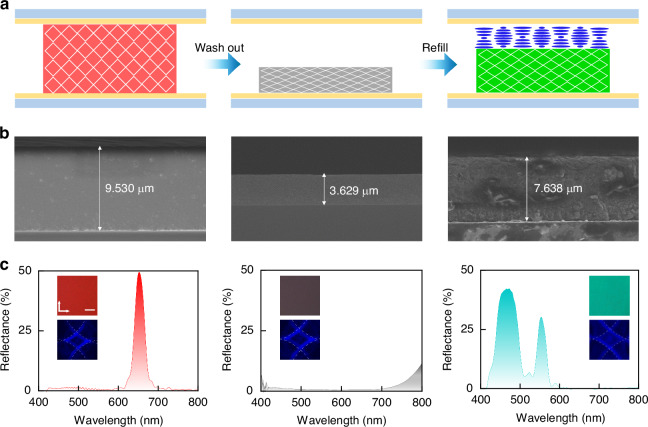
Fabrication and characterization of biphasic chiral photonic crystals. **a** Fabrication schematic: (i) PS-BPLC generation, (ii) monomer removal and template shrinkage, (iii) LH-CLC refilling to form the biphasic structure. **b** Cross-sectional SEM images of the PS-BPLC, polymer template, and refilled composite. **c** Reflection spectra with corresponding polarized optical microscopy (POM) images (top, scale bar: 50 µm; arrows indicate directions of the pair of polarizers) and Kossel diagrams (bottom) as insets for (i) the pristine PS-BPLC, (ii) the polymer template after washing, and (iii) the biphasic structure after refilling

The fabrication of a biphasic PC structure is achieved by washing out the unreacted monomers from a PS-BPLC and subsequently refilling the resulting template with an oppositely handed CLC. This process yields two stacked PCs: the original PS-BPLC and a newly formed CLC layer within the atmospheric gap created by network shrinkage. As illustrated in Fig. 2a, an RH-PS-BPLC with a 3D photonic lattice is initially prepared in a 10-µm cell. Following the solvent-based removal of nonreacted monomers, the polymer template underwent a significant thickness reduction from 9.53 to 3.63 µm (Fig. 2b, left and middle). Subsequent refilling with a LH-CLC mixture 1 only partially recovered the thickness to 7.64 µm (Fig. 2b, right). This irreversible contraction and the formation of flattened surfaces confirm the generation of an atmospheric gap, facilitating the formation of the second chiral PC—the LH-CLC layer.

The structural evolution during the wash-out-refill process is corroborated by corresponding reflection spectra. The pristine PS-BPLC exhibits an intense, narrow Bragg reflection band centered at 670 nm (lattice constant *a* is 299 nm), consistent with its uniform red texture observed under polarized optical microscopy (POM, Fig. 2c, left). After removing the unreacted monomers, the reflection band shifts into the ultraviolet region due to lattice contraction, resulting in the absence of a visible-range reflection band and a corresponding dark POM image (Fig. 2c, middle). As expected, the refilled cell displays two distinct reflection bands: a shorter-wavelength, broader band from the LH-CLC, and a longer-wavelength, narrow band from the recovered PS-BPLC. Notably, the PS-BPLC band blue-shifts to 560 nm (*a* is 241 nm) due to permanent lattice shrinkage, and the POM texture appears cyan from the superposition of reflections from both layers. The 130 nm blueshift of the reflective central wavelength is attributed to the shrinkage of the polymer network during the washing process. Meanwhile, the reflectance decreases from 42% to 30% because of the structural disorder introduced in the same process, which weakens the Bragg reflection. The opposite circular-polarization selectivity of these biphasic layers is verified by polarization-resolved reflectance measurements. Under right-handed circular polarization (RCP) illumination, only the narrow band from the PS-BPLC is detected, as its original helicity is preserved by the polymer network. Conversely, under left-handed circular polarization (LCP) illumination, only the broad band from the LH-CLC is captured, where the central reflection wavelength of 480 nm corresponds to a helical pitch of 317 nm. The structural colors observed under POM align with these spectral features (Fig. S1).

Kossel diffraction analysis further confirms the structures of the biphasic chiral PCs. A well-defined four-arc Kossel pattern, recorded from the initial PS-BPLC (inset, Fig. 2c, left), confirms its blue phase I (BPI) lattice. This pattern persists after monomer removal, indicating that the polymer template retains the BPI symmetry despite lattice shrinkage, which is verified by an enlarged diffraction angle. When the BP layer of the final biphasic structure faces the objective, the characteristic four-arc pattern remains observable from 120 °C down to room temperature (RT), demonstrating the enhanced thermal stability imparted by the polymer network (Fig. S2a). When the CLC layer faces the objective, the four-arc pattern from the underlying BPLC is visible above the CLC’s clearing point. Upon cooling, this pattern is gradually replaced by the characteristic concentric circle diagram of the CLC as it emerges, confirming the coexistence of both PC phases within the system (Fig. S2b).

### Near-field imaging

**Fig. 3:**
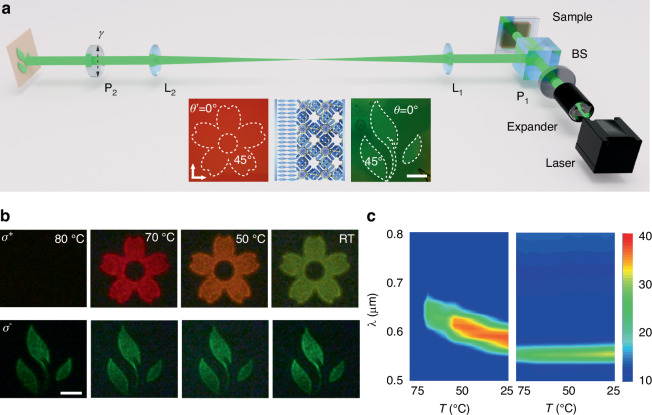
Near-field imaging in biphasic chiral photonic crystals. **a** Schematic of the reflective imaging setup. Insets show polarized optical micrographs of pre-aligned CLC (“flower” pattern, left) and BPLC (“leaves” pattern, right), with alignment directions labeled inside and outside the white dashed line marked patterns (scale bar: 550 µm). **b** Temperature-dependent near-field images. Under LCP illumination, the CLC (“flower”) channel exhibits a pronounced spectral shift at 580, 600, and 650 nm. Under RCP illumination at 560 nm, the BPLC (“leaves”) channel shows stable green reflectance (scale bar: 500 µm). **c** Central reflection wavelength of the biphasic chiral PC as a function of temperature for LCP

A 0°/45° binary patterned alignment *θx,y* is recorded in the initial PS-BPLC. After washing out the unreacted monomers and the atmosphere gap generated, a new pattern *θ’x,y* is photoaligned. The refilled LH-CLC still forms RH-PS-BPLC in the polymer template due to the polymer stabilization and maintains the *θx,y* alignment. While the CLC in the gap is left-handed and follows the alignment of the A scheme for the binary photopatterning is illustrated in Fig. S3. In this experiment, mixture 2 is adopted. The optical characterization of circular polarization interference is depicted in Fig. 3a. The setup probes the interference between two reflected circularly polarized components: one component is directly reflected by the chiral PC, while the opposite component undergoes a double pass through the rear PC layer after reflection from a mirror. The former beam carries a spatial Bragg-Berry phase of 2*θx,y* (from BPLC) or 2*θ’x,y* (from CLC), while the latter one carries a phase of −2*θx,y* (from BPLC) or −2*θ’x,y* (from CLC). The detailed information on the circular polarization interference is rigorously described by a Jones-matrix calculation (Supplementary Section 1)^31^, yielding a local intensity follows:

1 $$I(\gamma ,\theta )=2{\cos }^{2}(\gamma +2\theta )$$

For experimental demonstration, “flower” and “leaves” patterns are encoded as alignment patterns *θ’x,y* in the LH-CLC and *θx,y* in the RH-BPLC, respectively (insets, Fig. 3a). The resulting near-field images are characterized using a 4f imaging system (Fig. 3a). Briefly, a monochromatic beam, after passing through a polarizer, illuminates the biphasic sample. The two interfering circular polarization components are projected onto a screen via an analyzer and captured by a camera. Figure 3b shows the successfully decoded images, with both “flower” and “leaves” clearly visible. Notably, these images are observable by eye through an analyzer under monochromatic illumination, facilitating applications in optical encryption. When illuminated from the LH-CLC side with linearly polarized light, the “flower” image emerges only when the temperature is lowered below the clearing point. As the temperature further decreases to RT, the image color shifts from red to yellow, corresponding to a thermally induced blueshift of the photonic bandgap (Fig. 3b, images at 80 °C, 70 °C, 50 °C, and RT). When illuminated from the opposite (RH-BPLC) side, the “leaves” image is observed. The color and reflectance of this image remain stable against temperature variation due to the excellent thermal stability imparted by polymer stabilization. These observations are corroborated by spin-selective reflection spectroscopy (Fig. 3c). For LCP light, the reflection band shifts continuously from 650 nm to 580 nm with cooling, reflecting the thermal responsiveness of the LH-CLC. For RCP light, the central wavelength remains fixed, demonstrating the thermal stability of the RH-BPLC. Figure S4 shows the complementary image inversions achieved by adjusting *γ*. Thus, two independent near-field imaging channels are established for optical encryption. Notably, decoding the concealed information requires the correct combination of *σ*, *T*, *λ*, and *γ*.

### Far-field holography

**Fig. 4:**
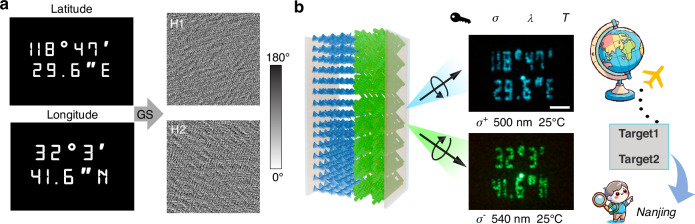
Spin-multiplexed holography. **a** Target information of longitude 118°47′29.6″E and latitude 32°3′41.6″N), along with corresponding photoalignment patterns (Hologram 1&2). **b** Scheme of the biphasic PC device for spin-multiplexed holography and decoded images at correct keys. The operation temperature is 25 °C, and the central wavelengths for CLC-generated holography and BPLC generated one are 500 nm and 540 nm, respectively (scale bar: 600 µm)

We further demonstrate biphasic PCs as far-field spin-decoupled holography, supplying two new separate channels for optical encryption. As a proof of concept, two phase diagrams corresponding to the geographic coordinates of Nanjing (Longitude: 118°47′29.6″E, Latitude: 32°3′41.6″N) are generated by the Gerchberg–Saxton algorithm. Then the obtained holograms (Fig. 4a) are encoded into the separate alignments of the bilayers. H2 corresponding to the latitude is encoded to the RH-BPLC, while H1 of longitude is recorded into the LH-CLC through a multi-step photoaligning process.

The decryption process can be envisioned as a symbolic “treasure hunt”, where the correct location remains concealed until specific keys are applied: *λ*, *σ*, and *T*. In this experiment, mixture 1 is adopted. At 55 °C, the LH-CLC is isotropic; under LCP illumination at both 500 nm and 540 nm, only a zero-order diffraction spot appears in the far field (Fig. S5). It is the same for 500 nm RCP light as the wavelength lies outside the photonic band of the RH-BPLC. In contrast, 540 nm RCP illumination falls in the photonic bandgap of the RH-BPLC, which successfully reconstructs the latitude. However, the whole encrypted information is still not retrieved successfully. At RT, as shown in Fig. 4b, incident 500 nm LCP light clearly reveals the longitude information (118°47′29.6″E), while incident 540 nm RCP light reconstructs the latitude information (32°3′41.6″N). The crosstalk between the holograms generated by light of opposite spins is negligible for the excellent spin selectivity of the biphasic layers. This specific architecture overcomes the constraint of conjugated phase modulation inherent in conventional liquid-crystal elements, thereby enabling spin-decoupled geometric phase modulation for multiplexed optical encryption.

## Discussion

The biphasic PCs are successfully fabricated using a straightforward washing-out-refilling strategy. This approach enables the stacking of two photopatterned PC layers with opposite handedness within a single cell. The resulting structure overcomes the intrinsic spin-wavelength coupling of conventional liquid crystal systems, allowing independent functionalization for orthogonal spin states and discrete wavelengths of incident light. Dual-channel optical encryption is thereby demonstrated in both near-field reflections and far-field diffractions. The thermal responsiveness of the CLC layer adds a further physical dimension to the decoding process. For near-field reflection, a binary patterned alignment technique (0°/45°) is introduced to establish two independent imaging channels; decryption here requires the precise combination of *σ*, *T*, *λ*, and *γ*. In the far field diffraction mode, holographic information remains concealed until the correct set of *σ*, *λ*, and *T* is applied. This biphasic design significantly enhances encryption complexity while retaining a simple and scalable fabrication route. Both the PS-RH-BPLC layer and the LH-CLC layer exhibit narrow Bragg reflection bands with a full width at half maximum (FWHM) in dozens of nanometers scale. Notably, the FWHM extends slightly along with the red-shift of the central wavelength. Among thermal tuning, adjacent wavelengths should be over the FWHM to make sure the spectral distinguishability. Due to the orthogonal spin selectivity of separate layers, generated near-field reflections and far-field diffractions by different layers are totally independent. Compared with traditional optical encryptions made by bulky optics, metasurface^32,33^ and LCs^34–38^, the proposed platform significantly enhances the unclonability of information entropy via introducing more degrees of freedom of light (e.g., spin, propagation direction, and near/far field). By introducing independent control over multiple photonic states-spin, wavelength, and polarization-coupled with an external thermal stimulus, our platform creates a vastly expanded and dynamically reconfigurable cryptographic dimension, thereby achieving a significantly higher level of encryption security.

Future improvements may include, (i) tailoring the decoding temperature by adjusting the composition of the refilled CLC; (ii) extending the Bragg reflection range by optimizing the thermal response of chiral dopants, adopting a lower birefringent LC host to reduce the FWHM and enlarging the number of selective channels; and (iii) integrating the structure with metasurfaces or diffractive optical elements to further expand functional versatility. The demonstrated applications, including thermally responsive anti-counterfeiting markers and spin multiplexed holography, illustrate the potential of biphasic chiral PCs for advanced information security. This platform may thus pave the way for next-generation optical encryption, secure displays, and multifunctional photonic devices.

## Materials and methods

### Materials

The BPLC is mixed with a host nematic LC HTG135200 (*n*_o_ = 1.5143, *n*_e_ = 1.7136, HCCH, China), the reactive monomers RM257 (NCLCP, China) and RM006 (NCLCP, China), the right-handed chiral dopant LC756 (NCLCP, China), a nonmesogenic crosslinker TMPTA (J&K Scientific Ltd., China) and the photoinitiator I-819 (J&K Scientific Ltd., China), with concentrations of 50, 22, 20, 5, 2.5, and 0.5%, respectively. The phase transition points for the BPLC precursor are: isotropic (ISO)-74 °C-BPI-68 °C-cholesteric (N*). Separately, two left-handed chiral liquid crystal (LH-CLC) mixtures were prepared for refilling by combining the host nematic HTW114200-050 (Δ*ε* = 10.9 at 1 kHz and 20 °C; Δ*n* = 0.222 and *n*_*e*_ = 1.729 at 589 nm, 20 °C. HCCH, China) with the left-handed chiral dopant S811 (NCLCP, China) at weight ratios of 69:31 (mixture 1) and 77:23 (mixture 2).

### Fabrications

The indium-tin-oxide glass substrates are activated by UV-Ozone and then spin-coated with 0.3 wt% photoalignment agent SD1 (NCLCP, China) dissolved in dimethylformamide (DMF, Sigma-Aldrich, USA). After being heated at 100 °C for 10 min, the two substrates are separated by 10-μm spacers and sealed with epoxy glue. The photo-aligned patterns are fabricated using a digital micromirror device-based microlithography system (NCLCP, China), and a multi-step partly-overlapping exposure process is performed to carry out the designed distributions accordingly (Fig. S6). Biphasic chiral PCs were fabricated via a stepwise “polymer-stabilization-wash-out-refill” procedure. First, the BPLC precursor was capillary-filled into photopatterned LC cells at a temperature above the clearing point (isotropic state). The filled cells were then slowly cooled at a rate of 0.2 °C min⁻¹ to promote the nucleation and growth of an ordered BPI texture. The obtained BPI state was subsequently stabilized by in situ photopolymerization under 405 nm irradiation (10 mW cm⁻², 60 s), yielding PS-BPLC films (Fig. S7). To generate the polymer template, the PS-BPLC films were immersed in acetone (Merck KGaA, Germany) for 6 h to remove unreacted monomers and other extractable components, followed by drying at 40 °C for 2 h. Finally, an LH-CLC was refilled into the template-containing cell, producing the biphasic chiral PCs within the same device architecture.

### Characterizations

The reflection spectra are recorded with a spectrometer (PG2000-pro, Ideaoptics, China). All the micrographs are captured at the reflective mode under a POM (Nikon 50i, Japan), and the Kossel diffraction is observed with a microscope (Olympus, BX51, Japan). The cross-section images are captured by a scanning electron microscope (SEM, TESCAN Brno s.r.o., Czech). Monochromatic beams are output by a supercontinuum laser (SuperK EVO, NKT Photonics, Denmark) and selected by a multichannel acousto-optic tunable filter (SuperK SELECT, NKT Photonics, Denmark). The near-field reflection setup (JCOPTIX, China) included a beam expander, polarizer, quarter-wave plate, beam splitter, and a 4f imaging system with two 150-mm lenses. A digital camera (EOS M, Canon, Japan) is used to capture the diffraction patterns. The temperature is precisely controlled with an LTS 120 hot stage (Linkam, UK).

## Supplementary information

Supplementary Information

## Data Availability

The data that support the findings of this study are available from the corresponding author upon reasonable request.
