# Supplementary material for: Multi-parameter enhanced optical encryption with biphasic chiral photonic crystals
Source: Light Sci Appl. 2026 Jun 4;15:266. doi: 10.1038/s41377-026-02360-z (PMC13237150; doi:10.1038/s41377-026-02360-z)
Supplement: Supplementary file 1 — Supplementary Information [file 41377_2026_2360_MOESM1_ESM.pdf]

Supplementary Information for: Multi-parameter enhanced optical encryption with biphasic chiral photonic crystals

Cheng Ouyang[1,†], Quanming Chen[1,2,†], Deiwei Zhang[1], Zhiyao Xie[1], Dan Luo[2,], Yan-qing Lu[1,*], and Wei Hu[1,*]*

C. Ouyang, Q. Chen, D. Zhang, Z. Xie, Y.-q. Lu, W. Hu

[1] National Laboratory of Solid State Microstructures, Jiangsu Physical Science Research Center, College of Engineering and Applied Sciences, Nanjing University, Nanjing 210023, China

Q. Chen, D. Luo

[2] Department of Electrical & Electronic Engineering, Southern University of Science and Technology, Shenzhen 518055, China

*Corresponding author: luod@sustech.edu.cn; yqlu@nju.edu.cn; huwei@nju.edu.cn.

†These authors contributed equally to this work.

Section S1: Jones-matrix calculation on the near-field circular polarization interference

The near-field images come from the interference between two orthogonal circular polarizations:

(1) the one directly reflected by the front chiral PC which carries a spatial Bragg-Berry phase of $2\theta_{x,y}$ (from front BPLC) or $2\theta'_{x,y}$ (from front CLC);

(2) the other reflected by the rear mirror which passes through the front PC layer directly and propagates 2 round trips in the rear one. It carries $-2\theta_{x,y}$ (from BPLC) or $-2\theta'_{x,y}$ (from CLC) and exhibits an opposite circular polarization.[1]

When a linear polarization with wavelength located in the bandgap of front PC incidences, the output field for the chiral biphasic-PC-mirror system is described by a Jones vector,

$$E_{\text{in}}\left(x,y\right)=\begin{bmatrix}E_{\text{L}}(x,y)\\E_{\text{R}}(x,y)\end{bmatrix}, \tag{S1}$$

where $E_{\text{L}}\left(x,y\right)$ and $E_{\text{R}}\left(x,y\right)$ are the complex amplitudes of LCP and RCP components, respectively. In this case, the Jones matrix of a mirror is given by,

$$M=\begin{bmatrix}0&1\\1&0\end{bmatrix}, \tag{S2}$$

which simply flips the handedness of circular polarization without spatially varying the phasefront. Let's take the LCP incidence as an instance. As the photoalignment pattern of front PC is depicted by θ, the selectively reflected component flips its spin and carries a Bragg-Berry phase of $+2\theta$, which can be described by

$$R_{\text{font}}(\theta)=e^{+i2\theta}\left|\text{L}\right\rangle. \tag{S3}$$

The orthogonal spin reflected by the mirror flips its spin, carries a Bragg-Berry phase of -2θ, and then is reflected by the mirror and flips its spin again. The variation during the total process is described by

$$R_{\text{mirror}}(\theta)=e^{-i2\theta}\left|\text{R}\right\rangle. \tag{S4}$$

Applying these operators to the incident field E_{in}, the circular polarization component reflected by the front PC is

$$E_{\text{front}}=\mathrm{e}^{+i2\theta}\begin{bmatrix} E_L \\ 0 \end{bmatrix}, \tag{S5}$$

while the orthogonal component reflected by mirror is depicted by

$$E_{\text{mirror}}=\mathrm{e}^{-i2\theta}\begin{bmatrix} 0 \\ E_R \end{bmatrix}. \tag{S6}$$

Their combination forms a total Jones vector as

$$E_{\text{out}}=\begin{bmatrix} E_{\mathrm{L}}\mathrm{e}^{+i2\theta} \\ E_{\mathrm{R}}\mathrm{e}^{-i2\theta} \end{bmatrix}. \tag{S7}$$

For simplification, we set an equal amplitude of 1 for both E_{L} and E_{R}, and the standard circular basis vectors are depicted by $|L\rangle=\frac{1}{\sqrt{2}}\begin{bmatrix} 1 \\ i \end{bmatrix}$, and $|R\rangle=\frac{1}{\sqrt{2}}\begin{bmatrix} 1 \\ \text{-}i \end{bmatrix}$. The S7 can be transformed to

$$E_{\text{out}}=\frac{1}{\sqrt{2}}\begin{bmatrix} \mathrm{e}^{i2\theta}+\mathrm{e}^{-i2\theta} \\ i(\mathrm{e}^{i2\theta}-\mathrm{e}^{-i2\theta}) \end{bmatrix}. \tag{S8}$$

After passing through a linear analyzer at angle γ, whose matrix is described by

$$P(\gamma)=\begin{bmatrix} \cos^2\gamma & \cos\gamma\sin\gamma \\ \cos\gamma\sin\gamma & \sin^2\gamma \end{bmatrix}. \tag{S9}$$

Then the reflectance (I) generated by the circular polarization interference is expressed as

$$I(\gamma,\theta)=2\cos^2\left(\gamma+2\theta\right), \tag{S10}$$

leading to a periodic bright-dark change along with the variation of (γ + 2θ). Above analysis suits for the case of RCP as well. It establishes the fundamental for the near-field circular-polarization interference.

Section S2: Design and fabrication of holograms

As shown in Fig. 6a, the DMD-based micro-lithography setup enables pixelated UV polarized exposing in a point-to-point manner. The resolution of photopatterning reaches 1.08 μm, which are determined by both the size of separate micro mirrors and the image reduction ratio of the optical system.

The flowchart in Fig. 6d describes the Gerchberg–Saxton algorithm phase retrieval process for generating the target longitude (118°47'29.6"E) hologram, which iteratively refines the phase function over 100 cycles. The full geometric phase hologram is composed of 18 sub-patterns (Fig. 6b), with three representative sub-patterns (corresponding to polarizer angles: 40°, 90°, 130°) shown in Fig. 6c. After exposures of all 18 sub-patterns accumulated, the overall phase diagram is recorded into the alignment layers. The LC azimuthal orientation guided by the alignment directly determines the Bragg-Berry (BB) phase encoded to the reflected light. The POM image in Fig. 6e verifies the successful fabrication of the target longitude hologram encoded in the BPLC layer.

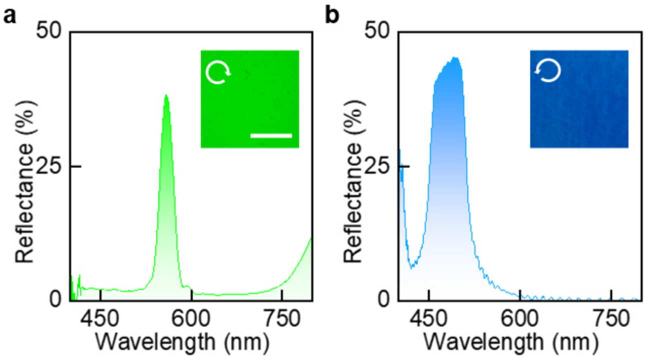

Fig. 1. Micrographs and reflection spectra of the biphasic PCs under: **a** RCP and **b** LCP illuminations.

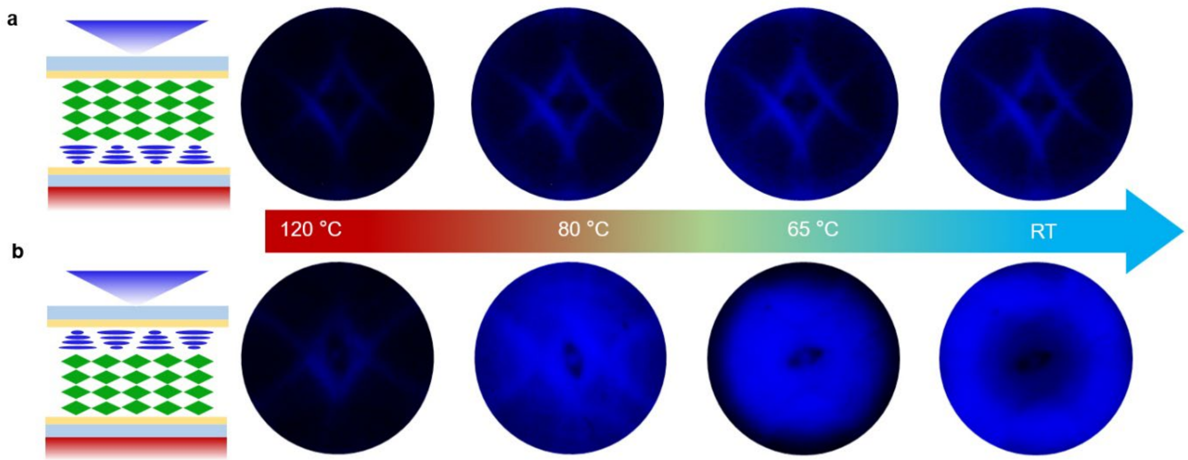

Fig. 2. The dependency of Kossel diffraction patterns on temperature for: **a** BPLC-up and **b** CLC-up conditions of the biphasic PCs.

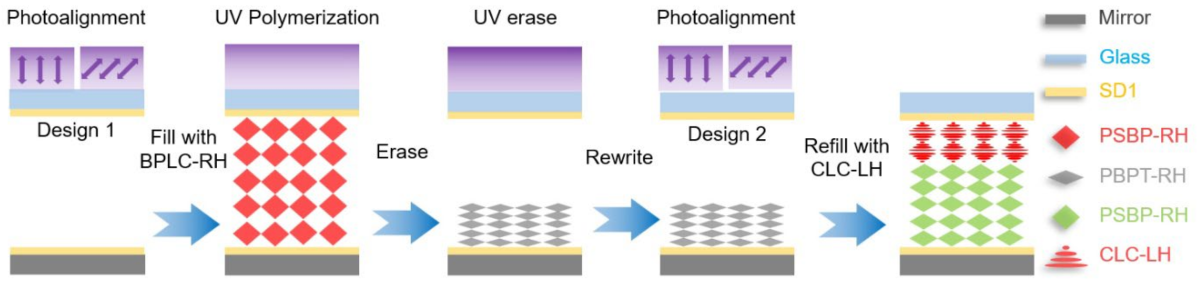

Fig. 3. The fabrication process of the bilayer differently photopatterned biphasic PCs.

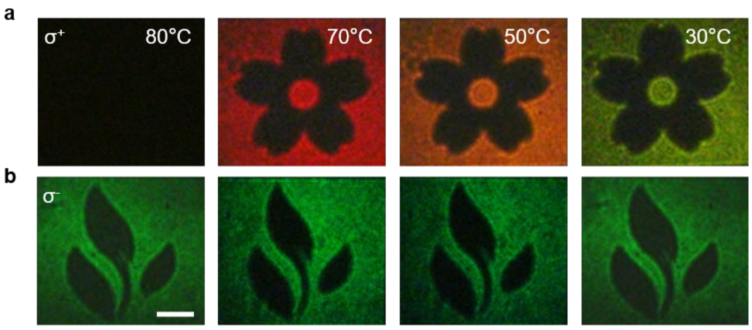

Fig. 4. Complementary versions of: **a** flower and **b** leaves as shown in Fig. 3 after rotating γ by 90° (scale bar: 500 μm).

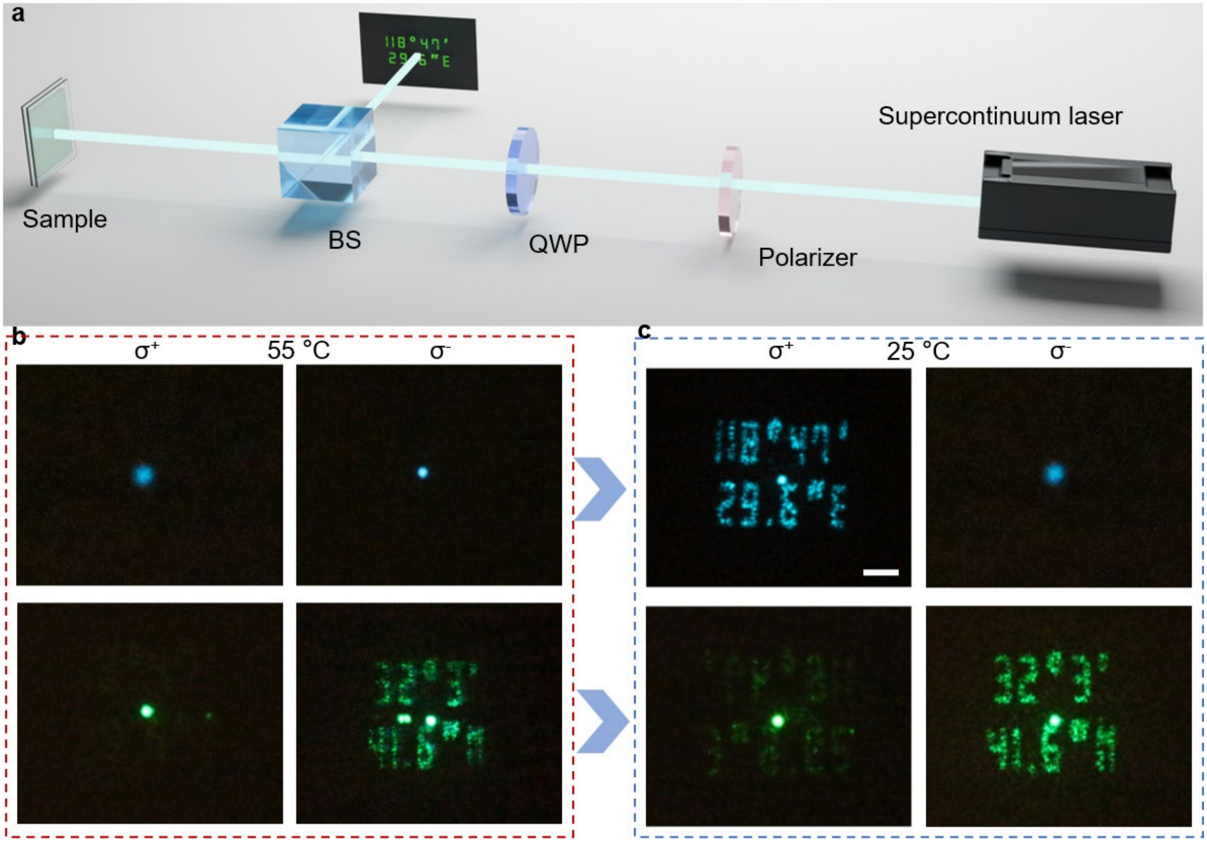

Fig. 5. a Optical setup for the holographic characterization. Spin and wavelength selected holography at **b** 55°C and **c** 25°C, respectively (scale bar: 600 μm).

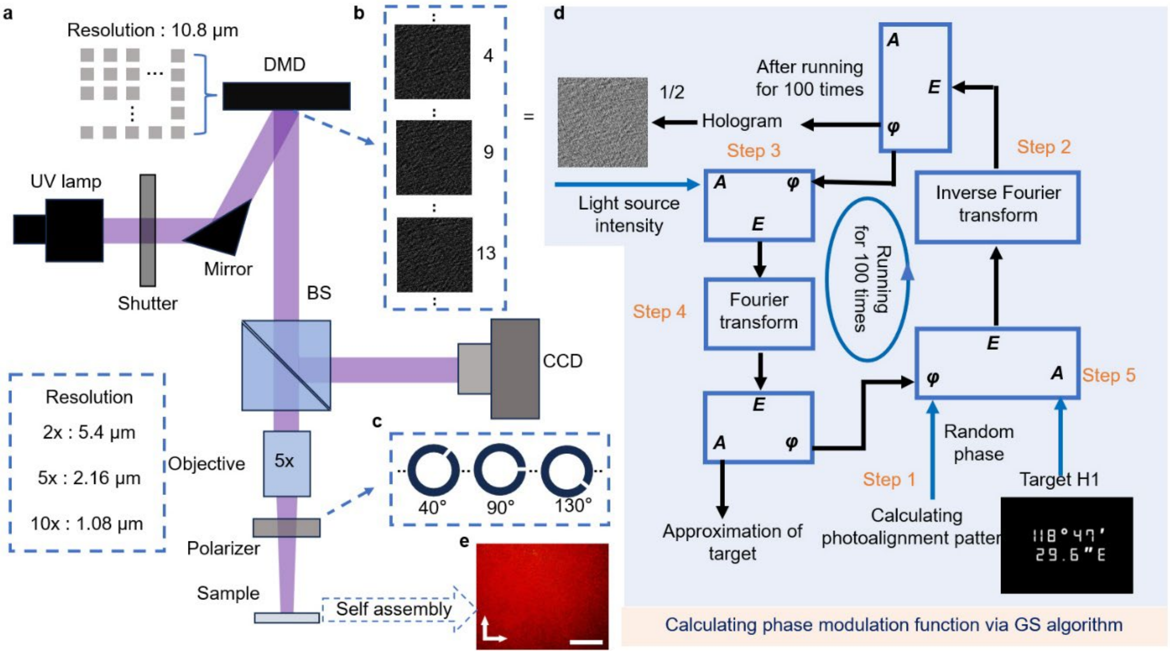

Fig. 6 a Schematic illustration for the DMD-based micro-lithography setup. **b** Three out of all 18 exposure sum-regions from a geometric phase hologram, with corresponding polarizer angles listed in **c**. **d** The flow chart for generating geometric phase holograms. A and φ indicate amplitude and phase of light field E, respectively. **e** The POM image of the hologram corresponding to the longitude 118°47'29.6"E (scale bar: 200 μm).

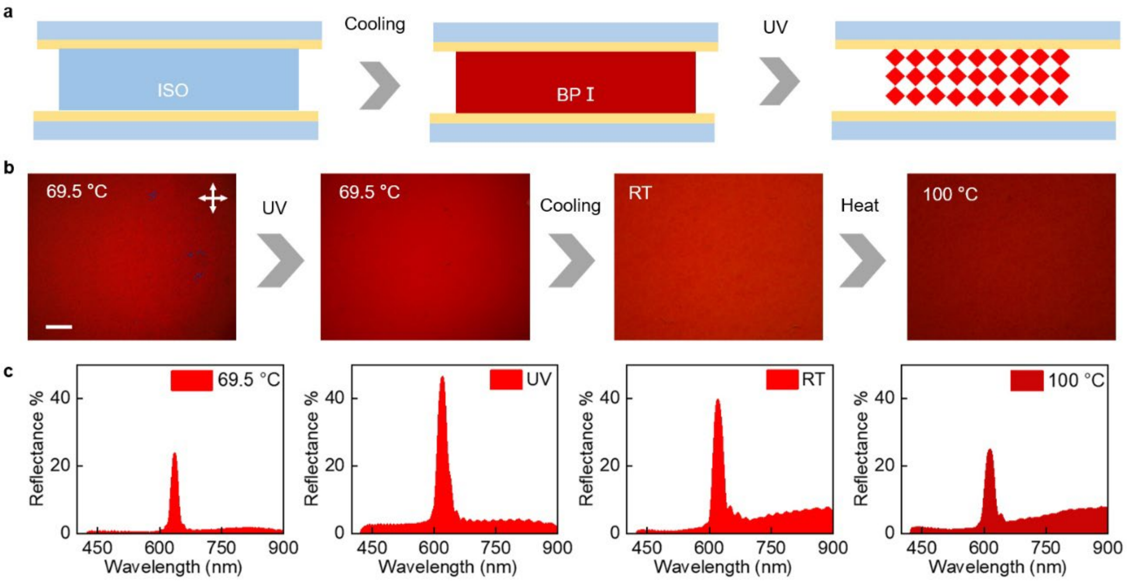

Fig. 7. Polymer stabilization and thermal-recycling processes. **a** Schematic illustration for the fabrication route of PS-BPLC. **b** POM images taken at 69.5 °C before/after UV curing, and those recorded at room temperature (RT) and 100 °C during thermal recycling (Scale bar: 200 μm). **c** Corresponding reflection spectra for states shown in (b).

References

1. Rafayelyan, M. & Brasselet, E. Spin-to-orbital angular momentum mapping of polychromatic light. *Physical Review Letters.* **120**, 213903 (2018).
